# Priorities for collaborative research using very preterm birth cohorts

**DOI:** 10.1136/archdischild-2019-317991

**Published:** 2020-02-06

**Authors:** Jennifer Zeitlin, Mariane Sentenac, Andrei S Morgan, Pierre Yves Ancel, Henrique Barros, Marina Cuttini, Elizabeth Draper, Samantha Johnson, Jo Lebeer, Rolf F Maier, Mikael Norman, Heili Varendi, the RECAP Preterm child cohort research group, Ulrika Ådén

**Affiliations:** 1 Université de Paris, CRESS, Obstetrical Perinatal and Pediatric Epidemiology Research Team, EPOPé, INSERM, INRA, F-75004, Paris, France; 2 EPIUnit-- Instituto de Saúde Pública da Universidade do Porto, Porto, Portugal; 3 Clinical Care and Management Innovation Research Area, Bambino Gesù Children’s Hospital, Rome, Italy; 4 Department of Health Sciences, University of Leicester, Leicester, United Kingdom; 5 Department of Primary & Interdisciplinary Care, Disability Studies, Faculty of Medicine, University of Antwerp, Antwerpen, Belgium; 6 Children's Hospital, University Hospital, Philipps University Marburg, Marburg, Germany; 7 Department of Clinical Science, Intervention and Technology, Karolinska Institutet, Stockholm, Sweden; 8 Department of Neonatal Medicine, Karolinska University Hospital, Stockholm, Sweden; 9 University of Tartu, Tartu University Hospital, Tartu, Estonia

**Keywords:** epidemiology, neonatology, neurodevelopment, patient perspective

## Abstract

**Objectives:**

To develop research priorities on the consequences of very preterm (VPT) birth for the RECAP Preterm platform which brings together data from 23 European VPT birth cohorts.

**Design and setting:**

This study used a two-round modified Delphi consensus process. Round 1 was based on 28 research themes related to childhood outcomes (<12 years) derived from consultations with cohort researchers. An external panel of multidisciplinary stakeholders then ranked their top 10 themes and provided comments. In round 2, panel members provided feedback on rankings and on new themes suggested in round 1.

**Results:**

Of 71 individuals contacted, 64 (90%) participated as panel members comprising obstetricians, neonatologists, nurses, general and specialist paediatricians, psychologists, physiotherapists, parents, adults born preterm, policy makers and epidemiologists from 17 countries. All 28 initial themes were ranked in the top 10 by at least six panel members. Highest ranking themes were: education (73% of panel members' top 10 choices); care and outcomes of extremely preterm births, including ethical decisions (63%); growth and nutrition (60%); emotional well-being and social inclusion (55%); parental stress (55%) and impact of social circumstances on outcomes (52%). Highest ranking themes were robust across panel members classified by background. 15 new themes had at least 6 top 10 endorsements in round 2.

**Conclusions:**

This study elicited a broad range of research priorities on the consequences of VPT birth, with good consensus on highest ranks between stakeholder groups. Several highly ranked themes focused on the socioemotional needs of children and parents, which have been less studied.

**What is already known on this topic?**

- More knowledge is needed on the long-term health, behavioural, emotional and social status of children born very preterm.
- There appears to be little improvement in long-term outcomes of children born very preterm despite improved survival and neonatal care within the last three decades.
- Knowledge is limited on the efficacy of postdischarge follow-up programmes and other services for children and their families.
- Collaborative data platforms using data from existing very preterm cohorts could optimise research on the long-term consequences of preterm birth.

**What this study adds?**

- Diverse stakeholders identified a broad scope of priority themes related to the consequences of very preterm birth that can orient collaborative research.
- There is good consensus on several high ranking priorities among a wide range of themes.
- Socioemotional needs of children and parents, which have been less studied in this population, are highly ranked by all stakeholder groups.

## Introduction

Every year between 1% and 2% of births are very preterm (VPT), occurring at <32 weeks of gestation, totalling over 50 000 babies in European Union countries.^1^ Improved survival over past decades has led to more VPT babies being discharged home from the neonatal intensive care unit (NICU). These children face higher risks of cerebral palsy, visual and auditory deficits, poor respiratory outcomes, impaired motor and cognitive ability and psychiatric disorders than children born at later gestations.^2–4^ While there are some reports of decreasing risks of cerebral palsy among VPT children,^5–7^ several recent meta-analyses and cohort studies have found that the prevalence of neurodevelopmental impairment has not changed and may even be rising.^2 3 8^ These studies call attention to the lack of progress in tackling the long-term consequences of VPT birth.

The need to promote research on the consequences of VPT birth was the motivation for the RECAP Preterm (Research on European Children and Adults Born Preterm) project, a European initiative to develop a research platform for VPT cohorts. Twenty-three cohorts from 15 European countries constituted over three decades are participating in this project to create the infrastructure, data dictionaries and harmonisation algorithms to facilitate collaborative research projects. As part of its development, the RECAP Preterm project will implement several demonstration projects to test the platform. This study sought to engage researchers from the participating cohorts as well as an external panel of stakeholders to guide the choice of the demonstration projects on the consequences of preterm birth for child and family outcomes up to 12 years of age. A secondary aim was to provide an overview of the current research concerns of stakeholders on the consequences of very preterm birth in childhood.

## Methods

This study implemented a modified two-round Delphi process with a multidisciplinary and geographically diverse panel of European stakeholders. The Delphi process is a formalised method for obtaining consensus, whereby participants respond to successive questionnaires that aim to identify common principles or proposals.^9 10^ It is used for multiple purposes, including determining common priorities for research.^11–14^ Responses are qualitative (free text comments) and quantitative (assigning ranks/scores). The Delphi process allows for anonymity, ensures an equal voice for all participants, provides feedback to the group to encourage iteration and interaction, and generates summary measures of agreement.^9 10^ Unlike some Delphi processes, we did not aim to eliminate themes or to achieve a shortlist of the most important topics. As such, a two-round process was considered sufficient.

**Figure 1:**
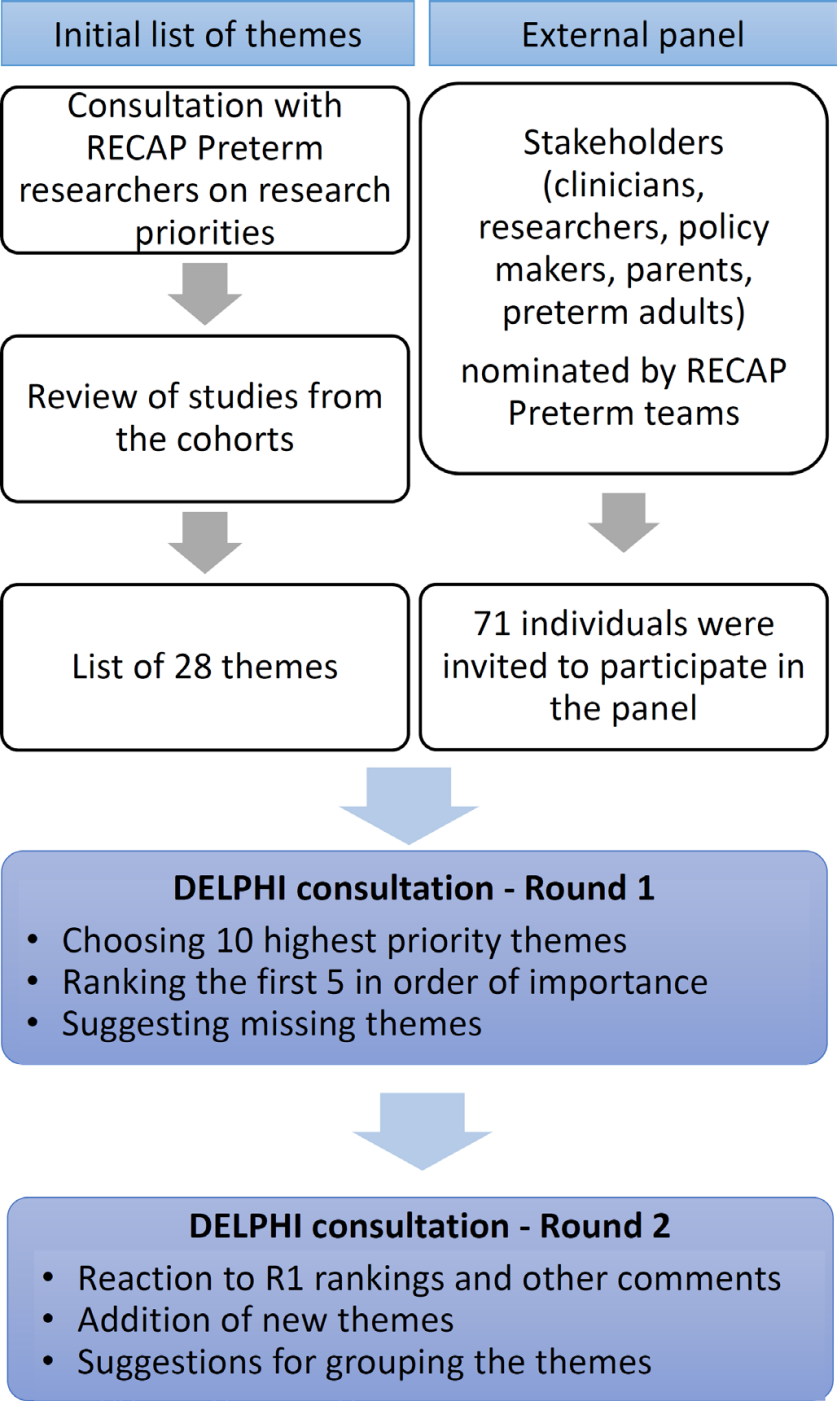
Methodology for the consultation process to identify research priorities on the consequences of very preterm birth.

The starting point for the Delphi was establishing a list of research themes concerning child and family outcomes up to 12 years (figure 1). While the RECAP Preterm project includes adult and child cohorts (listed in online supplementary appendix 1), our aim was to establish a research agenda using data from all cohorts, including recent cohorts that do not have long-term follow-up. The list of themes was derived through iterative consultation using online surveys with researchers from participating cohorts (11 participants from contemporary child cohorts, followed by 25 participants from all cohorts). We also reviewed the discussion sections of published cohort studies. Integrating quantitative evidence from other sources would have been of great interest, but was not considered feasible. Twenty-eight themes were defined using this process. Each theme was summarised in plain language for round 1 of the Delphi questionnaire (online supplementary appendix 2).

10.1136/archdischild-2019-317991.supp1 Supplementary data

Each cohort provided between three and six suggestions for Delphi panel members. Nominated candidates had to be external to the RECAP Preterm project, have good knowledge of preterm birth and represent diverse perspectives, including those of health professionals involved in the care of children born very preterm, researchers, policy makers (eg, health agency directors), parents, parent representatives and preterm-born adults. Parents and preterm adults were also identified with the help of The European Foundation for the Care of Newborn Infants (EFCNI), a parents’ association participating in RECAP Preterm. The questionnaire was in English, but responses could be in national languages with cohort representatives contacted to provide clarifications.

In round 1, panel members selected their top 10 priorities from the list of 28 themes, ranked their top 5 from 1 (highest) to 5 and identified missing themes. In round 2, we obtained feedback on the first-round rankings, asked whether newly suggested themes ranked in the top 10, and requested ideas for grouping themes and other comments. In the instructions, panel members were asked to select research themes needed to underpin clinical practice and/or health policy based on their own experience.

To analyse round 1 results, we created several summary scores: an average of rankings whereby the highest ranking theme was given a score of 10, the second highest given a score of 9 and so on. As only the top five were ranked, a score of 5 was given to non-ranked items in the top 10 and a 0 score was given to non-ranked items. We also counted the number of panel members ranking the theme in their top 10, 5 and 1. For round 2, we counted top 10 endorsements for new themes. Comments were analysed quantitatively (eg, number expressing agreement) as well as qualitatively to describe the panel’s opinions.

## Results

Of the 71 individuals nominated by the cohorts or the EFCNI, 64 (90%) participated in at least one Delphi round with 60 (85%) in each round (table 1). The panel included participants from 17 countries and multiple backgrounds, further classified into: (1) health professionals involved in the perinatal period, (2) health professionals involved in follow-up, (3) parents and preterm-born adults and (4) other.

**Table 1:** Characteristics of the external Delphi panel

Characteristics	Total	Round1/Round2	Health professionals in perinatal period	Health professionals involved in follow-up	Parents and preterm adults	Other
	n=64	n=60/60	n=19	n=19*	n=16*	n=*8* the star is misplaced
Country						
Austria	1	1/1	X			
Belgium	7	7/7	X	X	X	
Canada	2	2/2		X		
Czech Republic	1	1/1	X			
Denmark	1	1/1				X
Estonia	5	5/4		X	X	
Finland	2	2/2	X	X		
France	7	7/6	X	X	X	X
Germany	5	5/3	X		X	
Ireland	1	1/1			X	
Italy	5	5/5	X	X	X	X
The Netherlands	4	4/4	X	X		X
Norway	4	2/3	X	X		
Portugal	3	4/4		X	X	X
Spain	3	3/3			X	
Sweden	7	5/7	X	X	X	X
UK	6	4/6	X	X	X	
Discipline/Background*						
Obstetrician	4	4/4	X			
Neonatologist	17	14/17	X			
Nurse in neonatology	1	1/1	X			
Paediatrician	7	6/7		X		
Paediatrician subspecialist†	5	5/4		X		
Psychologist	7	7/7		X		
Physiotherapist	1	1/1		X		
Parent/Parent representative	11	11/10			X	
Adult born preterm	5	5/4			X	
Policy maker	4	4/3				X
Epidemiologist	3	3/3				X
Sociologist	2	1/2				X

*Three members were classified in two categories (parent/sociologist, parent/psychologist, sociologist/policy maker).

†Neurologist (n=2), endocrinologist (n=2), ophthalmologist (n=1).

All 28 themes in round 1 were rated in at least six top 10 lists and every theme was in at least one top 5 list (table 2). Despite the support for a broad range of themes, there was high agreement on a smaller set of themes. Themes with highest rankings (average score ≥3.5 and >50% top 10 scores) were: education of very preterm infants; care and outcomes of extremely preterm births, including ethical decisions; growth and nutrition; emotional well-being and social inclusion; parental stress and impact of social circumstances on outcomes.

**Table 2:** Ranking of themes by the external panel (round 1, n=60)

Theme	Average ranking*	Top 10 count†	Top 5 count†	Top 1 count†
Education of very preterm infants	4.6	44	18	3
Care and outcomes of extremely preterm birth, including ethical decisions	4.5	38	23	8
Growth and nutrition, including breast feeding	4.1	36	19	7
Emotional well-being and social inclusion	3.5	33	13	2
Parental stress	3.5	33	14	4
Impact of social circumstances on outcomes	3.5	31	15	6
Obstetrical and neonatal unit organisation and practices, including policies towards parents	3.2	29	14	5
Perinatal factors/treatments and long-term complications	3.1	28	15	4
Minor impairments and impact on learning and quality of life	3.0	27	11	1
Changes in disability status over time	2.8	27	10	3
Autism spectrum disorder and attention deficit and hyperactivity disorder	2.3	23	7	1
Cardiometabolic and pulmonary outcomes	2.3	20	11	2
Epigenetics/Genetic markers of poor outcomes	2.1	21	6	2
Motor development	2.0	20	8	0
Very preterm children from migrant families	2.0	22	7	0
Very severe fetal growth restriction	1.9	18	10	2
Intraventricular haemorrhage (IVH), including severe and less severe lesions	1.6	15	6	2
Necrotising enterocolitis (NEC)	1.6	16	4	1
Multiples	1.4	14	3	0
The wider environment (environmental and neighbourhood exposures)	1.2	10	6	2
Cerebral palsy (CP), including linking to CP registers	1.2	13	1	1
Maternal obesity and/or diabetes	1.0	10	3	0
Sub-fertility treatment	0.9	11	3	0
Validating predictive models of hospitalisation after discharge	0.9	9	3	1
Severe maternal morbidity during childbirth	0.9	10	3	0
Malformations	0.9	9	3	1
Older maternal age	0.9	9	3	0
Neurosensory impairments (blindness and deafness)	0.7	6	3	2

*See ‘Methods’ section for calculation of average rank.

†Number listing the theme as one of their top 10, 5 and 1 priorities.

**Figure 2:**
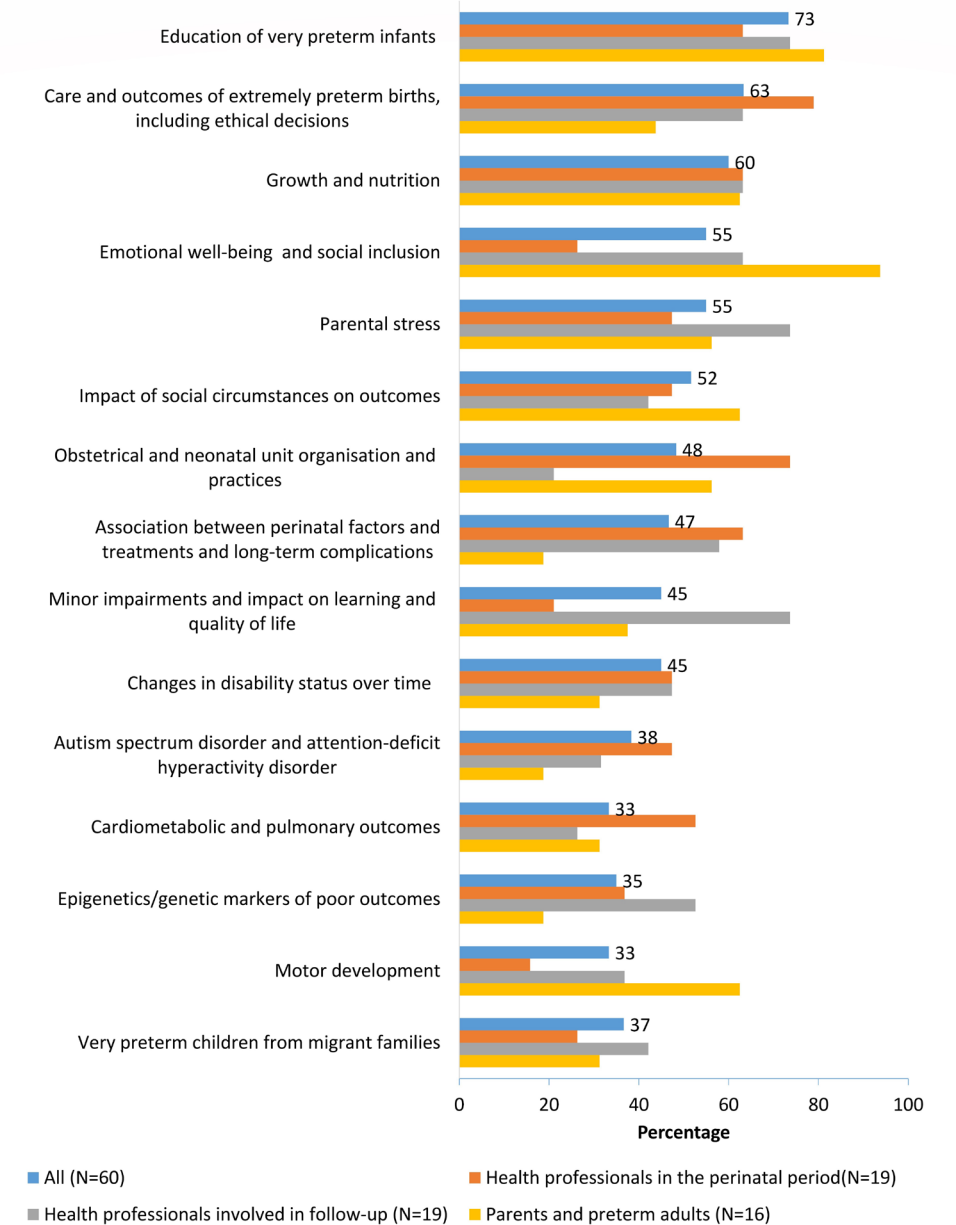
Themes most often selected in the top 10 priority lists by background of panel members (percentage of responses by group). Note: background category ‘other’ not included because of its small size and heterogeneous composition.

Among themes with a score ≥2.0 (corresponding to 20 or more top 10 endorsements), we compared top 10 ratings by the panel member background classification. This comparison showed good agreement on several highest ranking themes (figure 2). Some differences were notable, however, with parents being more interested in education, emotional well-being, social inclusion, the impact of social circumstances and motor development, while neonatologists and obstetricians expressed more interest in obstetric and neonatal organisation.

Eighteen respondents (30%) had no further comments about the themes. Others requested clarification that specific topics were included in an existing theme (eg, parental presence in the NICU in *obstetric and neonatal organisation*, maternal milk/breast feeding in *growth and nutrition*) and suggested ways to regroup themes. Some suggested themes were outside the study’s stated scope (adult outcomes, causes of preterm birth, issues specific to middle-income and low-income countries). Twenty new themes were suggested.

In round 2, 48 of 60 panel members commented on the rankings of which 30 were positive: “*Interesting and relevant, perceived as logical for me*” (Sweden, neonatal nurse). “*I feel that the themes with the highest scores really are appropriate*” (Spain, adult born preterm). “*The ranking reflects my view and I believe that this is a good starting point for prioritizing*” (Norway, psychologist). These are ‘*interesting results that are not unexpected’* (the UK, obstetrician). “*The list covered the most important topics. I agree with the order of priorities*” (France, neonatologist). Some endorsed the rankings even though they had different initial viewpoints "*after seeing the priority rankings and reading the description again, I think that these themes (care and outcomes of extremely preterm births and growth and nutrition) should be in the priority rankings*” (Portugal, parent representative).

Participants commented positively about the overlap between respondent groups: ‘*high correlation between the different subgroups indicates that this is a balanced composition’* (Belgium, physiotherapist) and this was seen to ‘*illustrate the concern, common to all participating groups, with long-term quality of life of the survivors’* (Portugal, paediatrician). The diversity in responses was valued: ‘*It is good that the top 10 ranking covers topics from different areas… This provides a broader picture of research related to very preterm children’* (Estonia, psychologist). Some respondents expressed regret that some topics were not more highly ranked: “*In general, I do agree with large parts of the priority ranking, although I would definitely rank topics such as ‘very severe fetal growth restriction’ or ‘NEC’ or ‘IVH’ significantly higher’* (Austria, neonatologist).

Other panel members expressed neutral opinions (ie, commenting on contrasts between stakeholders or suggesting alternative groupings). Six expressed more critical views related to missing themes and the overall process. One theme mentioned by two respondents was care in childhood: ‘*The monitoring and evaluation of care and outcomes over a long-term period (at least the whole preschool period) should have more votes’* (Italy, epidemiologist). Others criticised the scope “*Can we please try and PREVENT preterm birth…We have totally lost the relation with the factors that may cause prematurity*” (The Netherlands, policy maker) or the process itself: “*I am reluctant to prioritize themes—a grassroots type of approach that treats all ideas and initiatives as equal might be more appropriate*” (Germany, neonatologist) or “*Thinking about these issues in isolation is not, to me, as productive as discussing them in a broader group—so I struggle to devote enough time or thinking to the issues as they deserve*” (the UK, obstetrician).

In round 2, 15 new themes were ranked in the top 10 of 6 or more panel members, which corresponds to lowest ranking in the original list of 28 (table 3). No new theme received >27 votes which delineated the top 10 in round 1. New themes expanded the focus to economic costs and family organisation. Some themes overlapped somewhat with the original themes and overlap was noted by some panel members in the initial list. In round 2, we asked about regrouping or combining themes. The panel members’ replies were divergent, with some proposing to subsume individual topics into a few thematic categories and others insisting strongly that themes be kept specific. Given the absence of consensus, the themes were left in their original formulation (online supplementary appendix 3 provides the compiled list of themes with a top 10 ranking ≥6).

**Table 3:** New themes suggested by the external panel by number rated in top 10 in round 2 (n=60)

Theme	Top 10 count	%
Included in final list (≥6 top 10 votes)1		
Cognitive development	21	35
Economic consequences for family (including stopping/reducing work) and for society	14	23
Longitudinal studies over time looking at changes in care and outcomes	14	23
Parental mental health	13	22
Feeding problems	9	15
Retinopathy of prematurity	9	15
Language development, including multilingual education	8	13
Impact on the organisation of the family and other children in the family	8	13
Chronic lung disease	8	13
Quality improvement initiatives	7	12
Territorial and geographical dispersion/distribution of very preterm births—important for policy and prevention	7	12
Pharmacology/Medication /Pharmacokinetics of drugs	6	10
Microbiome studies	6	10
Role of primary care physicians in care of very preterm children	6	10
Long-term impact of extreme preterm birth on maternal outcomes (eg, later cardiovascular disease and diabetes)	6	10
Not included in final list (<6 top 10 votes)1		
Minor visual impairments	5	8
Hygiene in the neonatal unit	5	8
Cystic periventricular leukomalacia	4	7
Adolescent pregnancy	1	2
Older paternal age	0	0

1; Six corresponds to the lowest ranking theme in the original list of 28 themes.

The most highly rated themes informed RECAP Preterm’s initial research agenda on childhood outcomes, with three demonstration projects considered to be immediately feasible (care and outcomes of extremely preterm birth, including ethical decisions; growth and nutrition; impact of social circumstances on outcomes) and two areas for further development (education of very preterm infants; Parental stress).

## Discussion

This study elicited a broad range of research priorities covering the health, developmental, psychological and social consequences of VPT birth based on an initial consultation with very preterm cohort researchers and a modified Delphi process with an external panel of 64 stakeholders. There was robust consensus among panel members around a set of most highly ranked themes which were used to structure the research agenda on child outcomes within the RECAP Preterm platform. Highly ranked themes focused on medical management around birth, including ethical questions and the organisation of care, and on broader social issues, such as education and parents’ experiences. This study also revealed the diversity of stakeholder perspectives as reflected in some key differences in rankings by panel member background and in the high number of themes, 43, included in the top 10 lists of at least 6 panel members.

The study’s strengths were high participation rates from the external panel, geographic and disciplinary diversity and the participation of parents and very preterm born adults. While some consensus processes include users, patients or laypeople, in many cases respondents are clinicians or researchers only and response rates are often below 60%.^11–14^ One of the difficulties of the Delphi methodology is its complexity for laypeople and the ability to understand English constrained the people we invited to participate.^15^ In line with recommendations for carrying out Delphi exercises,^16 17^ the scope, objectives and intended outcomes of our study were predefined. However, some participants questioned the scope focusing on the consequences of VPT birth as opposed to the causes of prematurity. Furthermore, some panel members contested aspects of the methodology, including whether the technique lends itself to the level of reflection required. Finally, despite representation of a broad range of professionals who care for children born very preterm, not all specialties were included (ie, child psychiatrists, speech therapists), which may lead to under-representation of themes specific to these disciplines.

These results revealed a strong cross-disciplinary interest in the socioemotional repercussions of preterm birth. In particular, parental stress was ranked highly by all panel members, regardless of background. There is increasing awareness of the stressors on families linked to having a very preterm infant and the potential impact on children’s health and development,^18^ yet most research does not consider the topic of parental stress.^19^ Much less is also known about prognostic factors associated with the emotional well-being and mental health of the child.^20^ Finally, the strong interest in education, ranked in the top 10 of over 70% of panel members, suggests a need for an earlier and more comprehensive focus on the impact of VPT birth on life trajectories, a topic given visibility in the studies from cohorts of preterm born adults.^21^ Other top ranked themes were anchored around care in the period around birth, known to be determinant for mortality and morbidity and amenable to intervention, including the organisation of care and perinatal factors.^22–24^ While there is more research on these topics, this list highlights the limited knowledge about their long-term impact.

The range of themes in the final list illustrates the diversity of interests among stakeholders, the heterogeneous aetiology of VPT birth and the myriad ways that preterm birth impacts on child health and development and family function. Even among the highest ranking themes, professional background shaped priority rankings, with, for instance, less priority given to the organisation of care around birth by health professionals involved in follow-up and similarly less interest in emotional well-being by perinatal health professionals. Differences between professionals and parents also emerged, with the latter more interested in emotional well-being and social inclusion, education and growth and nutrition. The diversity of opinion in our panel was evident in questions about how the research themes should be grouped, leading us to retain themes as originally formulated without regrouping. These contrasting perspectives underscore the importance of including diverse opinions in consensus procedures and remind us the Delphi procedure is valuable for eliciting areas of common ground, and for sounding out the range of opinion and illuminating areas of difference.^16^

## Conclusion

Our study illustrated the broad span of research themes on the consequences of VPT birth in childhood considered to be priorities by stakeholders and identified several highly ranked themes with broad consensus to shape the RECAP Preterm research agenda. Initiatives to develop federated research constitute a valuable opportunity to involve the research community and other stakeholders in reviewing research needs.
