# Supplementary material for: Priorities for collaborative research using very preterm birth cohorts
Source: Arch Dis Child Fetal Neonatal Ed. 2020 Feb 6;105(5):538–44. doi: 10.1136/archdischild-2019-317991 (PMC7547907; doi:10.1136/archdischild-2019-317991)
Supplement: Supplementary data [file fetalneonatal-2019-317991supp001.pdf]

**Appendix 1**
**European very preterm cohorts participating in the RECAP Preterm platform**
*(for more information on the current European cohorts participating in the RECAP Preterm platform, including the number of inclusions, follow-up ages and data collected, see cohort profiles available on https://recap-preterm.inesctec.pt/cat/)*

| Cohort name | Country | Birth years |
|---|---|---|
| ACTION | Italy | 2003-2005 |
| AYLS | Finland | 1985-1989 |
| BEST-BLS | Germany | 1985-1986 |
| DNBC | Denmark | 1996-2002 |
| EPIBEL | Belgium | 1999-2000 |
| EPICE/SHIPS | Belgium, Denmark, Estonia, France, Germany, Italy, Netherlands, Poland, Portugal, Sweden, UK | 2011-2012 |
| EPICE-PT | Portugal | 2011-2012 |
| EPICURE 1 | UK and Ireland | 1995 |
| EPICURE 2 | UK | 2006 |
| EPIPAGE 1 | France | 1997-1998 |
| EPIPAGE 2 | France | 2011 |
| ESTER | Finland | 1985-1989 |
| Estonia 02-03 | Estonia | 2002-2003 |
| Estonia 07 | Estonia | 2007-2008 |
| ETFOL | Denmark | 1994-1995 |
| EXPRESS | Sweden | 2004-2007 |
| GNN | Germany | 2009-2010 |
| HeSVA | Finland | 1978-1985 |
| Pinkeltje/Lollipop | Netherlands | 2002-2003 |
| NTNU LBW Life | Norway | 1986-1988 |
| PEP | Norway | 1999-2000 |
| PIPARI | Finland | 2001-2006 |
| POPS | Netherlands | 1983 |



Zeitlin J, *et al. Arch Dis Child Fetal Neonatal Ed* 2020; 105:1–7. doi: 10.1136/archdischild-2019-317991

## Appendix 2 –
## Initial list of themes with descriptions sent in Round 1 questionnaire (some clarifications made after Round 1, noted in bold italics)

| **Influence of family, social and environmental factors on child outcomes** | |
|---|---|
| **1. Very preterm children from migrant families** | In some regions of Europe, up to 40% of very preterm infants have mothers who are migrants. Migrant families may live in difficult social circumstances and face language, communication and cultural barriers that limit their access to health and social services. In Europe, the characteristics and experiences of migrant populations are highly diverse depending on their country of origin, when they migrated and migrant policies in their host country. Another important issue for migrant children born preterm is how clinicians and researchers monitor their development and cognition since most clinical evaluations are designed for host-country children who speak only one language. |
| **2. Impact of social circumstances on outcomes** | Women with a low educational level or low incomes are more likely to have a preterm delivery. Very preterm children from socially disadvantaged families are also found to have worse neurodevelopment and health, although not in all studies or for all impairments. More understanding of these associations is needed to develop interventions to interrupt the transfer of health and social inequalities across generations. Importantly, early intervention in children from socially disadvantaged families may be particularly effective in improving developmental outcomes. |
| **3. Parental stress** | Having a very preterm infant can be stressful for parents. Knowledge is limited about how parental stress impacts on parent-infant interactions, child health and development as well as which interventions can effectively reduce stress for parents. Helping parents to cope with the stress of raising a very preterm child could improve the family environment and parents' ability to support their child. This could be an effective strategy for achieving better outcomes for very preterm children, especially with regards to their mental health and social inclusion. |
| **4. The wider environment** | The wider environment affects child health and development in many ways. Environmental exposures include pollution and other toxins as well as neighbourhood social and economic characteristics, such as social networks, crime rates, the availability of shops selling healthy food and fast-food restaurants. There are a growing number of studies on the impact of these environmental characteristics, but not specifically among very preterm children. Yet, children born very preterm may be particularly vulnerable to negative environmental conditions. |
| **5. Education of very preterm infants** | While many studies have investigated the cognitive, developmental and behavioural difficulties associated with very preterm birth, much less is known about how these affect performance and integration into school and how the school environment influences success in school. Within Europe, there is a large diversity in approaches to schooling, including age at school entry, policies related to staying behind a year and options for keeping children with difficulties in regular schools. This diversity provides an opportunity to learn from systems achieving good results. This theme covers children with moderate and severe motor |

| | |
|---|---|
| | and cognitive disabilities, who require specialised services at school, as well as children with minor difficulties. We will also study children with good school attainment to assess what promotes success in school. |
| **Growth and later development** | |
| **6. Minor impairments and impact on learning and quality of life** | Initial studies of the consequences of very preterm birth focused on major motor and neuro-developmental impairments, but children without these impairments are confronted with minor motor, cognitive and behavioural difficulties that impact on their health, learning and quality of life. Describing these difficulties and their consequences is essential for their prevention and treatment as well as for research on their causes. |
| **7. Growth and nutrition** | Finding optimal feeding and nutrition strategies, **including the promotion of breastfeeding,** represents a major challenge for the care of very preterm newborns. A wide range of policies currently exist, illustrating an absence of consensus. Research on this theme would aim to assess the impact of sub-optimal growth before and after birth (intra- and extra-uterine growth restriction) on longer term outcomes and to identify effective interventions to improve growth. Data from multiple cohorts could be used to develop robust tools for evaluating growth trajectories in very preterm children. |
| **8. Autism spectrum disorder (ASD) and attention-deficit hyperactivity disorder (ADHD)** | Preterm birth is a well-established risk factor for ASD and ADHD. The low prevalence of these conditions can make it difficult to study these disorders in small studies. There is also evidence that these disorders have a different clinical presentation and correlates in the preterm population. These results suggest that there may be differences in causes which has consequences for diagnosis, treatment and intervention. Combining data from several cohorts will provide more robust estimates of the prevalence of these disorders by gestational age and phenotypic profile, and will identify the specific risk factors and developmental mechanisms for ASD and ADHD among children born very preterm. |
| **9. Emotional wellbeing and social inclusion** | Studies on older children and adults born preterm find that emotional wellbeing and social inclusion are predominant concerns, yet much of the research on younger children focuses on physical health and development. This research theme would assess and evaluate existing data in cohorts of younger children on emotional disorders (panic disorders, anxiety and depression), as well as mental wellbeing and social participation. |
| **10. Motor development** | Many children born very preterm have mild to moderate motor problems that have an impact on their daily lives. Understanding the characteristics and the trajectory of these difficulties and relevant risk factors could inform physiotherapists and occupational therapists. Minor problems may also represent obstacles for learning in school and participation in social activities. Some cohorts have assessed motor outcomes at several ages and these could be combined to get a more complete picture of growth difficulties and their consequences during childhood. |
| **11. Cardiometabolic and pulmonary outcomes** | Children and adults born very preterm or very low birthweight have increased levels of several cardiometabolic risk factors including higher blood pressure and impaired glucose regulation, which can lead to increased risks of metabolic syndrome and type 2 diabetes in adulthood and possibly stroke and coronary heart disease. These children also |

| | |
|---|---|
| | have poorer pulmonary airflow than their peers born at term. The mechanisms underlying these associations are not well understood. Pooling data across cohorts increases the accuracy of risk estimates and could help to uncover additional risk and protective factors that could be targets for prevention. |
| **12. Changes in disability status over time** | While assessments of disability in early childhood are good predictors of later disability, many children change their disability status as they grow older. Some children are no longer considered to have a moderate or severe disability, whereas others are evaluated as moderately or severely disabled after a normal evaluation earlier in childhood. This research theme focuses on identifying the medical, social and healthcare factors leading to a change in disability status in order to improve prediction for clinical care and to understand what determines the clinical course of disability in this population. |
| **Perinatal care** | |
| **13. Care and outcomes of extremely preterm births, including ethical decisions** | Studying births at extremely early gestational ages is challenging due to their relatively low number. Lack of knowledge, particularly about longer term outcomes, has led to a range of attitudes in the management of these births by medical teams*,* with practices varying substantially between and within countries. A consortia approach would permit many questions around this topic to be tackled. These include methodological issues (how to assess gestational age, impact of stillbirths, definition of active management), better evaluation of the infants' potential for survival and survival without major disability (for example, by examining outcomes among births receiving active management), and evaluating the impact of perinatal management on rare (e.g. auditory or visual impairment) or subtle (cognitive or behavioural problems) outcomes later in childhood. |
| **14. Validating predictive models for hospitalization after discharge** | Understanding the risk factors for re-hospitalisation after discharge from the neonatal intensive care unit is needed for hospital staff to make the best discharge decisions. Discharging too early can expose the infant to adverse events, leading to re-hospitalization. However, prolonged stay in hospital lengthens exposure to risks associated with the hospital environment (for instance, nosocomial infections), may impede interactions between parents and the infant and can affect the hospital's capacity to admit other infants as well as their healthcare costs. European cohorts can contribute to defining generalisable predictive models to improve discharge decisions. |
| **15. Obstetrical and neonatal unit organization and practices** | The organization of healthcare services for very preterm infants is known to affect their survival free of morbidity. Factors within units like staffing adequacy, as well as hospital and regional level factors such as population density, geography and distances between hospitals, may have profound effects on hospital personnel and decision-making. The environment within the neonatal unit also differs across Europe based on the use of developmental care guidelines, including the number of children per room and **policies and facilities making it possible to facilitate** the presence of parents. The impact of different policies of care is unclear, particularly for longer-term child health. Combining information from different health systems could facilitate learning about best practices and optimal organization. |
| **16. Association between perinatal factors and** | There is a large literature investigating the associations between perinatal risk factors (such as medical complications at birth) and infant |

| | |
|---|---|
| **treatments and long term complications** | outcomes. This is also true for treatments and interventions used at birth and during the neonatal hospitalisation such as antenatal steroid administration, use of magnesium sulfate and hypothermia management. Short term effects of these treatments have been explored, but the impact on longer term outcomes remains unknown. Further research on health and development in children with these perinatal characteristics or receiving these treatments is needed to fully understand their impact and to develop optimal post-discharge healthcare strategies. |
| **Research on specific population risk factors** | |
| **17. Maternal obesity and/or diabetes** | Maternal obesity and diabetes are increasingly common in European countries and these are risk factors for very preterm birth. Research on term children finds that maternal obesity and diabetes are associated with specific developmental difficulties, such as language and other cognitive delays. Maternal obesity is also related to the child's future growth and in particular their risk of obesity in the future. These issues remain unexplored among very preterm children. |
| **18. Sub-fertility treatment** | Sub-fertility treatment increases risks of very preterm birth and is also linked to conditions such as preterm prolonged rupture of membranes which lead to preterm birth. Sub-fertility treatment is also a risk factor for congenital anomalies which are more common among children born very preterm. Given these links, it is important to describe the longer-term neurodevelopmental, physical and psychological morbidities related to sub-fertility treatment. |
| **19. Older maternal age** | In European societies, more women are having children after 35 and they are more likely than younger mothers to have pregnancy complications, such as very preterm birth. Investigating the specific risks associated with very preterm birth for children with mothers over 35 years of age could lead to improvements in care and counselling for parents. |
| **20. Multiples** | About one-third of very preterm infants are multiples. Multiple pregnancies have specific medical complications during pregnancy, which affect the children's later health and development and also pose specific challenges for parents. Areas for study include how to effectively breastfeed multiples, how to provide best care for children who may both require special services, and the impact of a co-twin's death - an occurrence in about one-quarter of deliveries - on maternal mental health. Multiple births are often excluded from population studies and there is less research on the long-term developmental outcomes of multiples compared to singletons in preterm populations. |
| **Research on neonatal morbidities or subgroups of preterm births defined by their medical characteristics** | |
| **21. Intraventricular haemorrhage (IVH) including severe and less severe lesions** | Severe bleeding in the brain (or severe intraventricular haemorrhage (IVH)) is one consequence of very preterm birth and leads to poor motor and neurodevelopmental outcomes. However, the health and developmental outcomes related to less severe bleeding are less well understood and some children with severe IVH develop normally. Preventive measures for IVH and how new imaging techniques can improve prediction and care are areas where research is needed. |

| | |
|---|---|
| **22. Necrotising enterocolitis (NEC)** | Necrotising enterocolitis (NEC) is a serious condition affecting very preterm newborns where tissues in the intestine become inflamed and start to die. There is a large variability in NEC prevalence across neonatal units and regions in Europe. Understanding this variability and whether practices from hospitals or regions with less NEC can be applied more broadly is an important area for research. Questions also exist on the long-term health of children who survived NEC. |
| **23. Very severe fetal growth restriction** | Restricted fetal growth is common among pregnancies ending in very preterm delivery. However, some fetuses experience very severe growth restriction with birthweights up to 4 or 5 standard deviations below what would be expected given their gestational age. Studies of fetal growth restriction have tended to group all infants with growth restriction together, but these very severe cases may have different causes and health and developmental outcomes. Combing data from cohorts could provide new information on these uncommon, but severe, situations. |
| **24. Severe maternal morbidity during childbirth** | Severe maternal morbidity is defined as a life-threatening condition affecting the mother during pregnancy, childbirth or after delivery (such as eclampsia or post-partum haemorrhage). Severe maternal morbidity occurs between 1 to 2% of deliveries in high-income countries, but is more common for very preterm deliveries. The simultaneous management of high-risk situations for both the mother and the infant at delivery may affect health outcomes for both of them. Whether the mother suffered severe morbidity has been neglected in research on the health and development of very preterm infants, but these mothers may be less likely to breastfeed and they face higher risks of depression, which may affect mother and child interactions. |
| **25. Malformations** | There is a higher frequency of malformations among very preterm infant than infants born at term. Children with serious anomalies are often excluded from analyses of outcomes as researchers search to identify prognostic factors linked solely to preterm birth. Children with minor congenital anomalies are sometimes excluded as well. This means that the specific needs of these children are neglected in research. As the type and severity of anomalies are very different, sufficient sample sizes (i.e. large enough studies) to be able to study specific, uncommon anomalies are difficult to achieve using national cohorts alone. |
| **26. Cerebral Palsy (with links to CP registers)** | Between 5 and 15% of children born very preterm are diagnosed with cerebral palsy (CP). Because these children represent a small proportion of all very preterm infants, studies have not focused specifically on this population. However, many research questions remain about the causes and characteristics of CP among very preterm infants and the later consequences for health and quality of life. Combining data from the very preterm cohorts would lead to a larger number of study subjects and make it possible to explore multiple research topics. Joint collaboration with the European network of CP registers (SCPE) could be explored. |

| | |
|---|---|
| **27. Neurosensory impairments (blindness and deafness)** | Children who are blind or deaf constitute less than 2% of very preterm infants, but these impairments have a major impact on their quality of life. Hence it is essential to understand their root causes for future prevention and consequences, and this can only be achieved by combining cohorts to get sufficient numbers of subjects. |
| **Other** | |
| **28. Epigenetics/genetic markers of poor outcomes** | Epigenetic modifications* such as DNA methylation are thought to be involved in mediating the relations of early-life stressors with health trajectories over the full life cycle, including into subsequent generations. Preterm birth potentially disturbs these modifications in irreversible ways, however, little is known about how modifications vary according to gestational age at delivery or about the interactions between very preterm birth and other early ex-utero exposures. ** changes to gene function which can be inherited, but that do not affect DNA sequences.* |

**Appendix 3 – Priority research themes from Round 1 and Round 2 (N=43)**

| Theme | Top 10 votes[1] |
|---|---|
| Education of very preterm infants | 44 |
| Care and outcomes of extremely preterm birth, including ethical decisions | 38 |
| Growth and nutrition, including breastfeeding | 36 |
| Emotional wellbeing and social inclusion | 33 |
| Parental stress | 33 |
| Impact of social circumstances on outcomes | 31 |
| Obstetrical and neonatal unit organization and practices, including policies towards parents | 29 |
| Perinatal factors/treatments and long term complications | 28 |
| Minor impairments and impact on learning & quality of life | 27 |
| Changes in disability status over time | 27 |
| Austism spectrum disorder and Attention deficit and hyperactivity disorder | 23 |
| Very preterm children from migrant families | 22 |
| Epigenetics/genetic markers of poor outcomes | 21 |
| Cognitive development | 21 |
| Cardiometabolic and pulmonary outcomes | 20 |
| Motor development | 20 |
| Very severe fetal growth restriction | 18 |
| Necrotising enterocolitis | 16 |
| Intraventricular haemorrhage, including severe and less severe lesions | 15 |
| Multiples | 14 |
| Economic consequences for family (including stopping/reducing work) and for society | 14 |
| Longitudinal studies over time looking at changes in care and outcomes | 14 |
| Cerebral Palsy (CP), including linking to CP registers | 13 |
| Parental mental health | 13 |
| Sub-fertility treatment | 11 |
| The wider environment (environmental and neighbourhood exposures) | 10 |
| Maternal obesity and/or diabetes | 10 |
| Severe maternal morbidity during childbirth | 10 |
| Validating predictive models of hospitalization after discharge | 9 |
| Malformations | 9 |
| Older maternal age | 9 |
| Feeding problems | 9 |
| Retinopathy of prematurity | 9 |
| Language development, including multilingual education | 8 |
| Impact on the organization of the family and other children in the family | 8 |
| Chronic lung disease | 8 |
| Quality improvement initiatives | 7 |
| Territorial & geographical dispersion/distribution of very preterm | 7 |

| | |
|---|---|
| births – important for policy & prevention | |
| Neurosensory impairments (blindness and deafness) | 6 |
| Pharmacology/medication /Pharmacokinetics of drugs | 6 |
| Microbiome studies | 6 |
| Role of primary care physicians in care of very preterm children | 6 |
| Long term impact of extreme preterm birth on maternal outcomes (e.g. later cardiovascular disease and diabetes) | 6 |

**NOTE: the threshold of 6 corresponds to the lowest-ranking theme in our original list of 28 themes**



Zeitlin J, *et al. Arch Dis Child Fetal Neonatal Ed* 2020; 105:1–7. doi: 10.1136/archdischild-2019-317991
